# A new era for optic pathway glioma: A developmental brain tumor with life-long health consequences

**DOI:** 10.3389/fped.2023.1038937

**Published:** 2023-03-24

**Authors:** David A. Walker, Kristian Aquilina, Helen Spoudeas, Chiara Pilotto, Hoong-Wei Gan, Lisethe Meijer

**Affiliations:** ^1^ Emeritus Professor Paediatric Oncology, University of Nottingham, Nottingham, United Kingdom; ^2^ Department of NeuroEndocrinology, Great Ormond Street Hospital, London, United Kingdom; ^3^ Pediatric Clinic, ASUFC Santa Maria Della Misericordia, Udine, Italy; ^4^ Kinderoncologie, Prinses Máxima Centrum Voor Kinderoncologie BV, Utrecht, Netherlands

**Keywords:** optic pathway hypothalamic glioma, childhood, treatment selection, health outcomes, vision loss, endocrine late effects

## Abstract

Optic pathway and hypothalamic glioma (OPHG) are low-grade brain tumors that arise from any part of the visual pathways frequently involving the hypothalamus. The tumors grow slowly and present with features driven by their precise anatomical site, their age at presentation and the stage of growth and development of the host neural and orbital bony tissues. Up to 50% of optic pathway glioma arise in association with Neurofibromatosis type 1 (NF1), which affects 1 in 3,000 births and is a cancer predisposition syndrome. As low-grade tumors, they almost never transform to malignant glioma yet they can threaten life when they present under two years of age. The main risks are to threaten vision loss by progressive tumor damage to optic pathways; furthermore, invasion of the hypothalamus can lead to diencephalic syndrome in infancy and hypopituitarism later in life. Progressive cognitive and behavioural dysfunction can occur, as part of NF1 syndromic features and in sporadic cases where large bulky tumors compress adjacent structures and disrupt neuro-hypothalamic pathways. Persistently progressive tumors require repeated treatments to attempt to control vision loss, other focal brain injury or endocrine dysfunction. In contrast tumors presenting later in childhood can be seen to spontaneously arrest in growth and subsequently progress after periods of stability. These patterns are influenced by NF status as well as stages of growth and development of host tissues. The past two decades has seen an expansion in our understanding and knowledge of the clinical and scientific features of these tumors, their modes of presentation, the need for careful visual and endocrine assessment. This influences the decision-making surrounding clinical management with surgery, radiotherapy, chemotherapy and most recently, the potential benefit of molecularly targeted drug therapy. This article, based upon the authors' clinical and research experience and the published literature will highlight advances in approach to diagnosis, the established role of vision loss as justification of treatments and the emerging evidence of endocrine and neurological consequences that need to be incorporated into judgements for case selection for therapy or observation. Consideration is given to the current state of biological evidence justifying current trials of new therapies, the genetic studies of the NF1 gene and the potential for new approaches to OPHG detection and treatment. The outstanding health system priorities from the perspective of children, their parents and health system commissioners or insurers are discussed.

## Introduction

Optic pathway hypothalamic glioma (OPHG) are a group of low-grade developmental tumors of the brain that can arise anywhere along the visual pathways from the optic nerves to the optic radiations as well as involving the adjacent hypothalamus and surrounding limbic structures. These tumors classically present in early childhood (under the age of eight years). Up to 50% are associated with the inherited cancer predisposition syndrome neurofibromatosis type 1 (NF1), which usually presents earlier in life at less than five years of age. From the perspective of children with NF1 up to 20% can present with OPHG. Overall, sporadic and NF1 associated OPHG account for 3%–5% of childhood brain tumors. They seldom metastasise within the central nervous system and almost never systemically. Long term survival into adulthood can be expected in over 80%. NF1, as a genetic cancer pre-disposition state, places individuals at increased risk of specific low-grade and malignant tumors throughout life. These lifetime risks influence treatment selection justifying minimal use of radiotherapy and avoidance of DNA mutating drugs such as alkylators, wherever possible. Furthermore, the risk of vision loss requires careful justification for the use of drugs with toxicities linked to hearing damage or other neurological toxicities (1, 2).

The detection and management of OPHG pose significant challenges for the wide variety of practitioners seeing children (3). Their deep midbrain, central location makes the majority unsuitable for surgical resection, without the risk of significant visual, endocrine and/or cognitive and behavioural consequences (4). Scientific progress in the past decade has identified targetable cellular growth pathways, which have opened up the opportunity for trials of innovative therapies (5). This article will address the following questions:

• How do OPHG present clinically and can we accelerate diagnosis?
• How do you select children for treatment and monitor its benefit and toxicity?
• What are the risks of vision loss
• What are the risks of neuro-endocrine deficiencies?
• How will the new clinical knowledge influence clinical practice?
• What are the trial questions under current study?
• What are the outstanding questions from patients and families and health care providers?
• What is the emerging biological evidence for current and future trials?
• What are the outstanding questions from the patients' and families' perspectives?

## How do OPHG present clinically and can we accelerate diagnosis?

**Figure 1:**
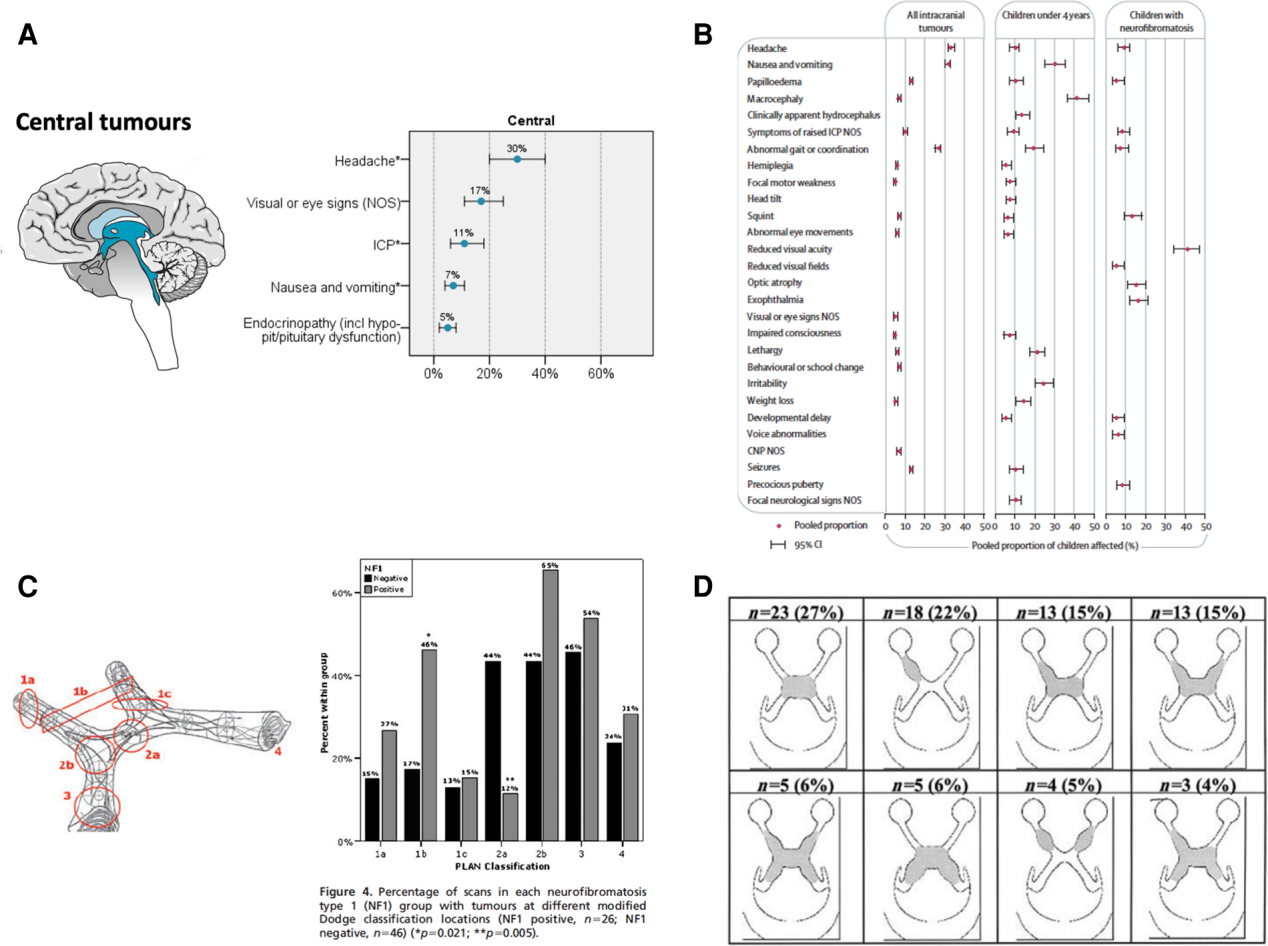
(**A**) Symptomatology of supratentorial/midline (central) tumors of childhood (3). (**B**) Comparison of brain tumor symptomatology for those with and without NF1 (11). (**C**) Comparison of anatomical distribution of OPHG between sporadic and NF1 types using the Modified Dodge Classification/PLAN Score (42). (**D**) Anatomical distribution of NF1 OPG in the multi-centre NF1 clinic cohort (84).

OPHG can present with:

• signs and symptoms of impaired visual function due to optic nerve damage the nature of which is related to the precise anatomical site of nerve involvement along the visual pathways. Nystagmus due to poor visual acuity or focal mid brain abnormality can occur;
• acute hydrocephalus requiring urgent cerebro-spinal fluid (CSF) diversion, particularly when the tumor or an associated cyst fills the third ventricle;
• proptosis due to retrobulbar optic nerve tumor displacing the eye forward;
• disturbances of growth and sexual development patterns due to disruption of afferent and efferent hypothalamic signalling;
• diencephalic syndrome due to hypothalamic tumor involvement in the first two years of life causing an extreme form of metabolic disturbance characterised by impaired weight gain with preserved growth in length /height, hyperactivity, hypermetabolism, persistent vomiting and an eye movement disorder (See Figures 1A, 1B).

Many of these presentations occur in the first five years of life from effects of growing tumor affecting the hypothalamic control of endocrine, metabolic and neuro-behavioural functions affecting longitudinal growth, weight gain, sexual development, cognitive and emotional functioning. Identification of a child with these presenting symptoms or signs requires parents (6), carers and practitioners to be aware, vigilant and curious to select children in a timely way for the key diagnostic tests for tumor diagnosis. Subsequent neurodevelopmental and endocrine assessments are required to delineate the degree of hypothalamic disorder.

## OPHG and NF1

Where NF1 has been established as a diagnosis by family history or observation of classical café au lait patches and other features of NF1; regular visual surveillance together with growth, puberty and developmental monitoring in the first five years of life, is recommended (1, 2). Brain imaging is increasingly being used as a screening/surveillance test to detect those at risk of progressive growth abnormalities and visual loss, especially if compliance with vision testing is sub-optimal. The benefits of screening with brain imaging in NF1, remains to be proven as many structural abnormalities of the optic pathways fail to progress and lead to vision loss, furthermore spontaneous tumor regression can occur. On the other hand, children can present with large tumor with minimal symptoms on surveillance. These situations parallel the challenge of detecting neuroendocrine signalling disturbance in NF1 in early life (7, 8), where GH excess syndromes in NF1 (6) which appear to spontaneously evolve to GH deficiency are increasingly reported in the youngest infants. Specific mutations within the NF gene are now recognised to be associated with the risks of optic nerve glioma development at specific developmental stages (1, 9) (See Figures 1C, 1D).

It has recently been established that raising awareness of early signs and symptoms of brain tumor in childhood amongst the public and health professionals can accelerate diagnosis of brain tumors; though growth/puberty abnormalities and thirst dysregulation remain poorly recognised symptoms by practitioners and the public (10). The HeadSmart programme identified age-stratified and NF status-stratified symptom checklists which have been published (11) and trialled with the public and health professionals. They have been shown to be acceptable for selection and rejection of patients for brain scanning. Their widespread use in the hands of the public and professionals has been associated with accelerating diagnosis of childhood brain tumor in the UK national health systems (3, 12, 13). However, tumors in the central region of the brain including OPHG, currently have the longest total diagnostic interval (3). Taken together, a clinical diagnosis of NF1 presents an opportunity for enhanced precision in predicting the risk, or early detection, of OPHG as a pathway to select young children for sight-preserving and neuro-endocrine evaluation strategies at an early stage.

## How do you select children for treatment and monitor its benefit and toxicity?

**Figure 2:**
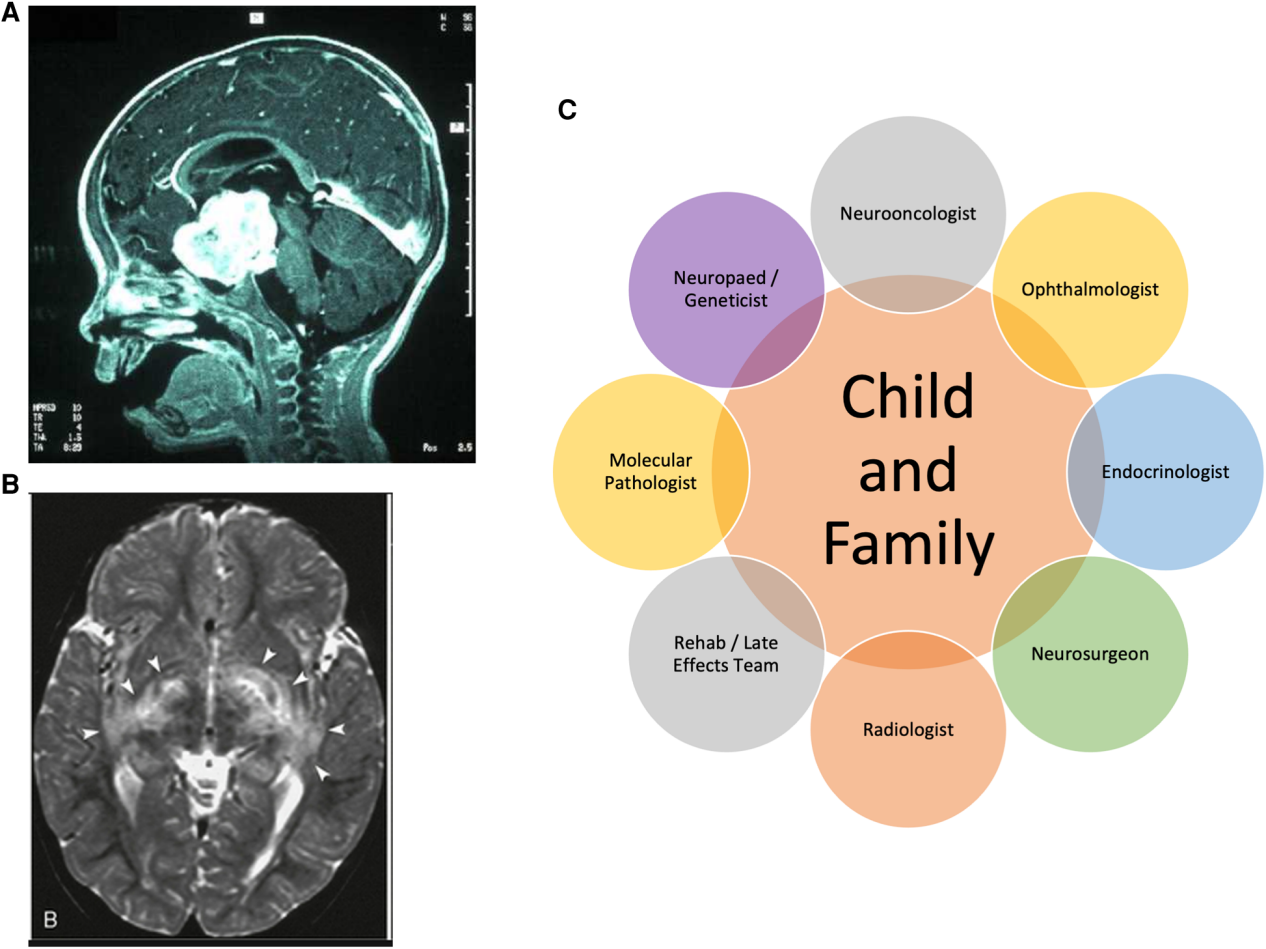
MRI scan of typical (**A**) sporadic hypothalamic and (**B**) multi-focal NF1 OPHG involving posterior radiations; (**C**) clinical specialisms involved in the OPHG multidisciplinary team.

Typically, the results of the brain scan make the diagnosis. The co-existence of clinical features of NF1, assessment of visual function, growth parameters, neurodevelopmental, endocrine and metabolic status/risk provide the key elements for consideration of treatment or observation. The European trials used a standardised age and NF1 stratified algorithm for case selection of medical treatments and radiotherapy (Figures 2A, 2B).

### Multi-disciplinary team assessment

It is recommended that all cases should be considered by the paediatric neuro-ophthalmic and neuro-oncology multi-disciplinary team and to these should now be added neuroendocrine and neurodevelopmental expertise (14). A key element of the clinical consideration is the role of neurosurgery for biopsy, management of raised intra-cranial pressure and consideration of tumor debulking (see below). A range of genetic mutations have been described converging on the MAPK/ERK regulatory pathway and contributing to functional activation of the pathway. The overwhelming majority are low grade histology with molecular characteristics defined in the recent WHO classification including what used to be described as pilocytic astrocytoma (PA), pilomyxoid astrocytoma, diffuse low-grade glioma and an adult variant of anaplastic pilocytic astrocytoma; rarely, higher grade gliomas occur in this anatomical region and need to be identified (5, 15).

Historically, multi-disciplinary teams had not specified that ophthalmologists, endocrinologists, neurodevelopmentalists or geneticists should be mandatory members for case discussion. As the treatments evolve under clinical trials, visual outcomes are now specified as primary outcome measures, requiring ophthalmologists to be central to decision-making and outcome measurement. It can be anticipated that, for children who often demonstrate occult endocrine presentations or evolving consequences of both disease and therapies, lifelong endocrine follow up will be required (16). Similarly, specialist genetics clinics now often manage children with NF1, especially where there are complex features and detailed genotyping offers risk assessment for OPHG development. The clinical perspectives of these disciplines are of great importance given the multiple problems associated with NF1 across all ages (2). The specialists with particular expertise in non-surgical therapy are the paediatric oncologists and radiotherapists whose role is to weigh the potential benefit of their anti-tumor therapies against the genetic and age-stratified risk of vascular (moya moya), endocrine, neurological toxicities and the risk of second tumors. The high survival rates for OPHGs make these judgements of particular importance (Figure 2C).

### Selecting cases for observation vs. treatment

*Diencephalic syndrome:* There is general agreement that infants presenting with diencephalic syndrome due to hypothalamic astrocytoma require drug treatment directed at reducing the tumor's metabolic activity and continued growth (17). Chemotherapy with vincristine and carboplatin or vinblastine monotherapy has been extensively used and the parameters of the hypermetabolic syndrome [in which GH excess may play a part] can be expected to be reversed by such treatments (17, 18). Serious neurological toxicity has been reported where tumor response is dramatic (19). As nutritional failure is a presenting feature, intensive nutritional management is needed in parallel with anti -tumor treatments (20). Even with attempts to treat such cases, the metabolic and neurological challenges are such that brain injury, spontaneous haemorrhage, surgical complications or acute neuroendocrine disruption can lead to life threatening complications. For those who survive lifelong neurobehavioural, neuroendocrine disturbances, as well as visual, hypothalamic and developmental consequences, can be expected.

### Indications for (immediate) surgical intervention

Modern clinical practice requires tumor tissue to be examined histologically and molecularly. In NF1, it is still justifiable to omit biopsy if there is any risk of surgery adding to vision loss, endocrine or neurological toxicity. In sporadic cases, biopsy is needed to ascertain both histological and molecular phenotype, especially if a child is to be entered in a clinical trial using targeted therapy. Management of hydrocephalus is also indicated where appropriate. The selection of cases for consideration of resection/debulking of hypothalamic tumors is an area of particular debate (4, 21, 22). While a significant proportion of the tumor infiltrates optic pathways and the hypothalamus, and is therefore unresectable without further harm, most OPHGs also contain exophytic components and cystic elements. Resection of exophytic tumor into the third ventricle or frontal lobes may be effective at reducing tumor size rapidly with minimal surgical risk. Cystic components exert high mass effect and are not generally responsive to chemotherapy. Drainage or fenestration of large cysts, or implantation of an indwelling reservoir, may be useful in supporting the benefits of chemo- and radiotherapy. Some tumors also have large posterior extensions, leading to symptomatic brainstem compression. In practice, the main difficulty lies in identifying the normal hypothalamic tissue radiologically and intra-operatively. Intra-operative MRI is useful to obtain a tailored resection with maximal safety (23). Although some series have advocated early and extensive resections, it is not clear that clinical outcome is improved in the long term (24, 25). The balance of risks between a large operative procedure that may itself cause hypothalamic injury but substantially reduce tumor bulk, and the long-term compressive effects of a large tumor on central structures is not known and needs further study. Similarly, the timing of major surgical interventions, and specifically whether surgery should be considered early after diagnosis or only after radiation and/or several cycles of chemotherapy have failed, is unclear. Specialist and multidisciplinary post-operative care and continuous endocrine and neurodevelopmental rehabilitation is needed after surgical resection. A recent institutional series of OPHG identified surgery of whatever type to be associated with risks of posterior pituitary endocrine failure in nearly 60% of cases (21, 26).

*Proptosis:* This presentation occurs with tumors arising in the optic nerve in the retro-orbital space. When presenting in the first two years of life, vision may be threatened or lost. If optic atrophy is present, vision recovery with chemotherapy will be limited by the established loss of nerve function. If vision is preserved and the main consequences are cosmetic, then differential growth of the orbit and tumor may reduce the severity of the proptosis in the first five years of life. The only surgical option is resection of the optic nerve for cosmetic reasons, if the eye is blind and the proptosis is disfiguring, leaving the eye *in situ*.

### Chemotherapy

The young children with OPHG (<5 years) have been offered treatment with chemotherapy as primary treatment over the past three decades. The drugs used have focused predominantly upon two drug classes: platinum agents: carboplatin/cisplatin and vinca alkaloids: vincristine/vinblastine (17, 27, 28). They were selected for their low mutagenic toxicity profiles, they can be administered as a day case in fractionated doses and have predictable toxicities. Intravenous administration is required for both drug classes and is associated with the risks of bone marrow suppression with neutropaenia, immunodeficiency, thrombocytopaenia and the need for blood transfusion. Carboplatin was found to be associated with significant drug reactions in up to 20% when given over prolonged periods (29, 30). Renal and auditory toxicities are important to watch for, but infrequent with carboplatin; they are predictable and more common with cisplatin. Vincristine is much less toxic to the bone marrow than vinblastine and was primarily selected for early trials for this reason. However, its prolonged pharmacological half-life (∼5 days) causes cumulative peripheral and autonomic neuropathy when used on a weekly schedule. There have been reports of vision loss associated with such neuropathies (31, 32). Using a drug, administered in neuropathic doses, to reverse a neuropathy seems unwise, just as is the use of ototoxic drugs where vision is already compromised/threatened. Adopting a four-weekly schedule to minimise the risk of vincristine neuropathy would seem a reasonable precaution. Monotherapy with carboplatin has comparable outcomes (33) for tumor control. Monotherapy with vinblastine is less neurotoxic but more marrow toxic (34) than vincristine. There is increasing experience in the use of monotherapies as primary therapy in OPHG. It is unclear whether speed of tumor response is comparable to combination therapies. Reports of irinotecan and bevacizumab in relapsed patients has been associated with improvements in vision (17). The optimal duration of therapy has not been determined. The use of these drug regimens ranging from 12 to 18 months have been reported. The age at treatment onset may be a key variable, given the tendency for tumor to spontaneously arrest in growth after 5–8 years of age. Tumor regrowth during adolescence is reported but not fully studied. Tumors have usually not been reported as progressive during adulthood. How these active and quiescent periods reflect age- and maturation-dependent key growth periods driven by hypothalamic hormone/neural signalling seems an important, and as yet, unexplored, future research question.

### Radiotherapy and its consequences

Radiotherapy has been used and has a stronger track record for controlling visual deterioration than chemotherapy but is known to cause impairments of local tissue growth of skull and brain tissue especially in very young children (35, 37). Radiotherapy is largely contra-indicated in NF1 because of the risk of secondary malignant tumor development within radiation fields (36). Radiotherapy involving the hypothalamic structures and adjacent carotid arteries carries additional risks of moya moya phenomenon of the carotid arteries (39–41). The risk of second tumors is lower for sporadic cases than for cases associated with NF1. Both carry the risk of (meningioma) and malignant tumor development such as Glioblastoma Multiforme (GBM). In NF1 the exaggerated risk of malignant peripheral nerve sheath tumors after radiotherapy is well recognised (38).The development of proton therapy with its more contained fields of treatment offers reduced risk of off-target radiation dosing with as yet unknown benefit on cognitive or endocrine function (37). Current practice is to defer radiotherapy until after one or more drug treatments have been tried, and been seen to have failed (37).

## What are the risks of vision loss?

*Visual development:* OPHG presentation during infancy and in pre-school age children is at a time where vision testing can restricted by their [in]ability to cooperate with visual acuity and field testing. Children's vision is in the process of developing to maturity during specific age- and time- dependent windows, and the brain's capacity to interpret the quality of image they experience which changes as their brain matures. This limits precision of early baseline vision assessment. Precise anatomical classification of tumors on imaging offers a prediction of the risk of bilateral vision loss (42) (See Figures 1C, 1D). Optical coherence tomography, measuring retinal fibre layer thickness, is being evaluated as a tool to detect early signs of optic nerve injury and its correlation with risks of, and actual, vision loss in young children (43). MRI studies of visual tracts with fractional anisotropy are also under evaluation as an imaging tool to predict visual loss (44).

### Can vision be improved or saved?

**Figure 3:**
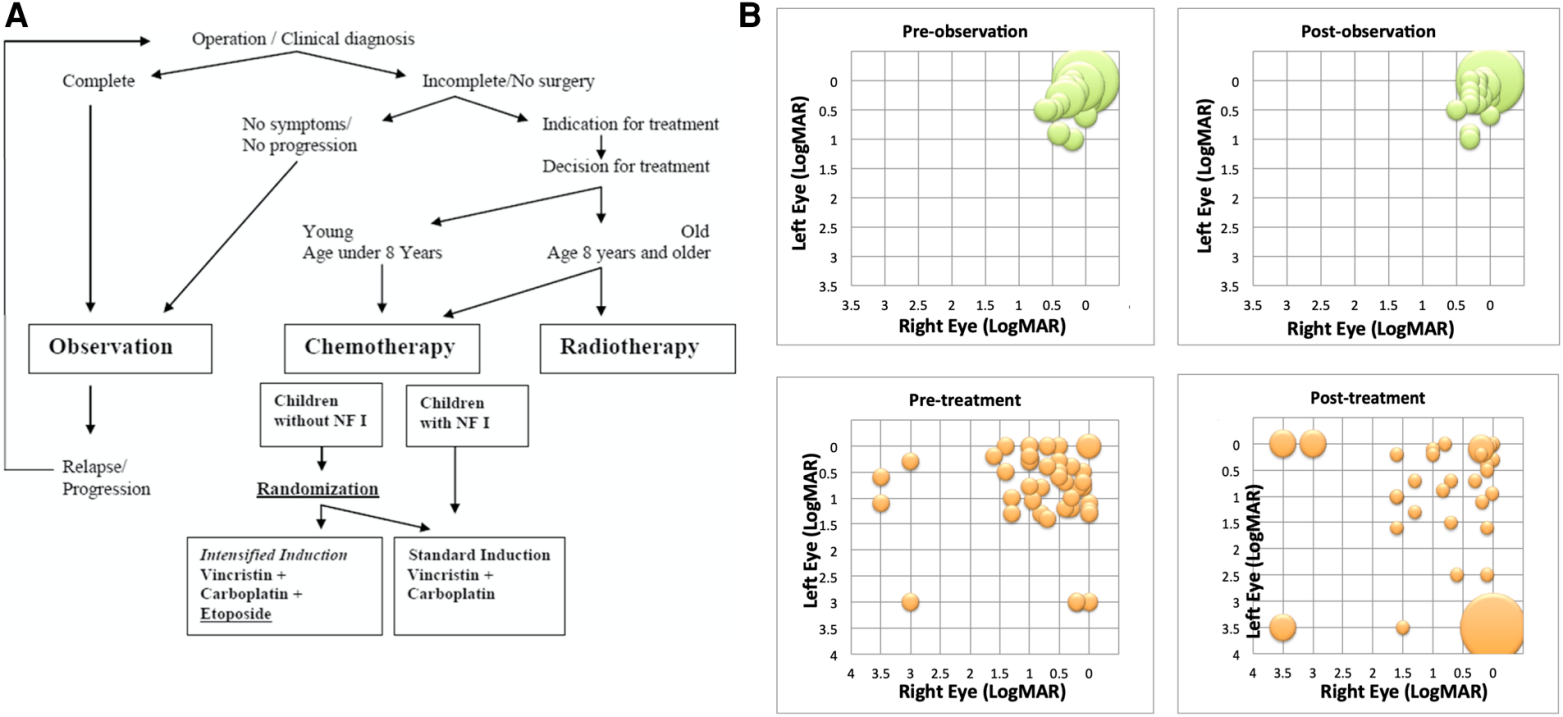
(**A**) Patient selection criteria for observation vs. treatment in SIOP LGG 2 (004 randomised trial (17). (**B**) Comparison of LogMAR visual acuity results from SIOP LGG 2004 workshop comparing pre- and post- bilateral visual acuity for observation (top green graphs) and treatment (lower orange graphs) with vincristine and carboplatin in patients with NF1 (46).

A recent systematic review failed to identify sufficient published information to reliably report the impact of treatments on visual outcomes (45). This has been studied in limited cohorts of children with NF1 and reported by US and European investigators (46, 47). The conclusions are influenced by the way their study cohorts were recruited. The US study was a multi-institution study cohort. It had a lower median age at diagnosis and reported only patients who were treated. The European study was trials-based and had an older median age and an observation arm and reported outcomes after “immediate therapy” and “therapy after observation” (48) (See Figure 3). Taken together, the following conclusions about visual outcomes can be drawn. Case selection at diagnosis has a big impact on visual outcomes. The European trial cohort had greater standardisation of case selection for treatment vs. observation than the institutional cohort where all reported, were treated. Despite these differences both studies showed overall, only 20%–30% of children experience improvement in vision with chemotherapy treatments. About 40%–50% experience stability of vision, whilst the remainder experienced deterioration in vision despite therapy. There was no clear correlation between imaging evidence of tumor response and visual outcomes. Those who are observed initially and seen to lose vision under observation, have a better chance of subsequently retrieving vision with therapy, compared to those treated immediately with more advanced vision loss and symptomatology at presentations. Specifically, those presenting with bilateral vision loss, multiple visual symptoms and optic atrophy seldom experience improved vision after therapy. The neurophysiological explanation for these observations has focused upon the rarity of spontaneous regression, whether the tumor is truly congenital and whether neuronal loss is related to local pressure effect or loss of trophic signalling between neurones and glia (49). Bevacizumab has been reported to improve vision in patients being seen to lose vision under observation (50). Standardisation of methods for measuring and recording imaging and vision outcomes have been developed to standardise selection of patients for treatments (2). Currently, the primary concern about vision loss due to tumor progression is used to justify commencement of treatments, a powerful motivating factor in the minds of parents. To date, apart from diencephalic presentations, endocrine status and late outcomes have not been widely used as a trigger for considering treatment or observation within trials.

### Seeking evidence to support selection of cases for observation vs. treatment

**Figure 4:**
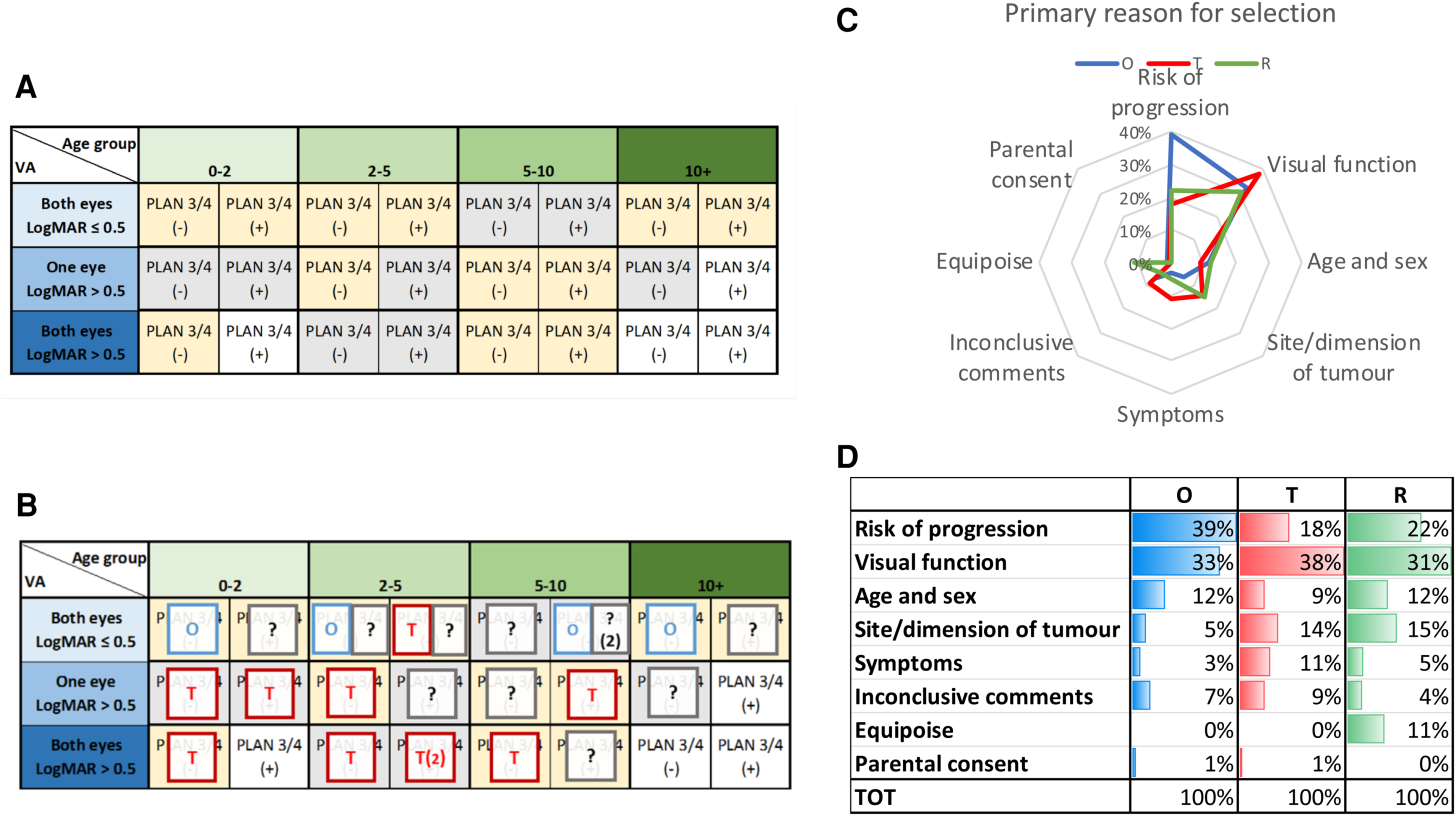
(**A**) A matrix of patient characteristics including visual acuity (LogMAR scores for one/both eyes), PLAN stage ¾ +/− (optic radation involvement) and age at diagnosis, (**B**): consensus (>70%) voting for 25 NF1 OPHG patient histories reported within the matrix identifying cases selected for initial observation (**O**), treatment (**T**) or? randomisation (?). (**C**) Spider plot of primary reason for consensus judgement for O,T & R. (**D**) Table of clinical reasons supporting strategy selection for O,T & R (51).

An international consensus survey was conducted using clinical and imaging information from children with OPHG associated with NF1 who were entered into SIOP LGG 2004 trial (51) (See Figure 4). These cases were presented in a questionnaire format to experienced international physicians (*n* = 98) from the full range of specialities involved in the design of clinical trials of therapy for OPHG. For each case they were offered the opportunity to observe, treat or randomise within a trial from a matrix of 25 cases structured by anticipated risk of tumor progression determined by unilateral or bilateral visual loss, age of the child and anatomical characteristics of the tumor. This consensus survey and its qualitative analysis of supporting comments identified that there was more than 70% agreement (consensus) on the selection of 14 out of 25 cases for observation or treatment. In 11/25 scenarios, however, the respondents did not reach consensus and considered them suitable for a randomised comparison of observation vs. treatment to determine the best course in future practice. The respondents identified the importance of as much detail as possible about the visual and neurological status of children in the period leading up to diagnosis and strategy selection, further supporting the justification for observation before treatment.

## What are the risks of neuro-endocrine deficiencies?

A single institution cohort of children with OPHG (*n* = 166), studied over 30 years has reported a 20-year overall survival (OS) of 81.0%, and progression-free (PFS) and endocrine event-free survival rates (EEFS) of 47.2 and 20.8%, respectively. Growth Hormone deficiency (GHD) affected 40.3%, followed by central precocious puberty (CPP, 26.0%), gonadotropin (GnD; 20.4%), TSH (13.3%), and ACTH (13.3%) deficiencies (16, 26). These develop hierarchically. Central precocious puberty (CPP) was associated with future gonadotrophin deficiency. Posterior pituitary dysfunction occurred in 57.9% after surgery involving biopsy or shunt procedures and was associated with 6/13 deaths in the whole cohort. In this cohort, half (50.2%) of surviving children were worryingly obese, with later risks of metabolic syndrome, and other life-limiting consequences including type 2 diabetes. Endocrine deficits ascribed to radiotherapy ranked growth hormone deficiency as the greatest risk followed by ACTH deficiency, insulin resistance and gonadotrophin deficiency. Endocrine Event Free Survival (EEFS) declined up to 15 years after diagnosis, with hypothalamic involvement of tumor being implicated more than radiotherapy in early onset endocrinopathy. GHD surprisingly increased in later treatment eras when radiotherapy was used less frequently (26).

90 children in this cohort were diagnosed aged <3 years and followed for 40 years, they are reported separately (16). Endo-metabolic dysfunction was reported in 58.7%, the main factor contributing to this risk was a clinical presentation with diencephalic syndrome, followed by tumor involvement of hypothalamus, the use of radiotherapy and surgery. These studies suggest a biphasic pattern of detecting endocrinopathy; at diagnosis, as a consequence of tumor damage, and after treatment, as a result of delayed damage from the tumor's continued impact and/or its treatment.

## How will the new clinical knowledge influence clinical practice?

**Figure 5:**
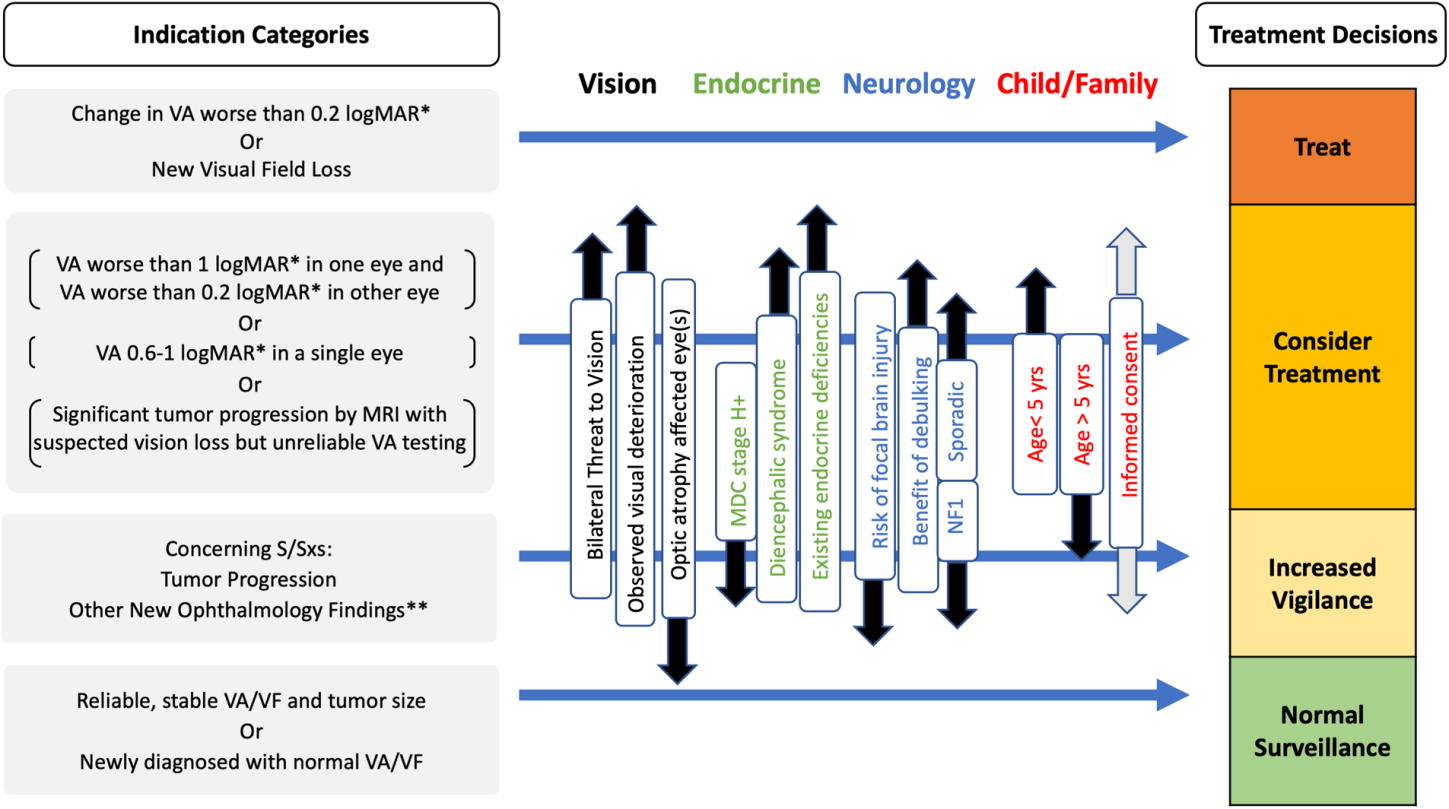
Evidence-based multi-disciplinary factors to be considered for selection of treatments (surgery, chemotherapy or radiotherapy) vs. observation in OPHG of infancy and childhood in OPHG Adapted from (3, 46, 79).

This information about endocrine outcomes is newly described and needs to be integrated with multi-disciplinary decision-making, outcome assessment and discussion of the benefits and risks of therapy as well as targeted individualised endocrine remediation in clinical practice. OPHG clinical complexity poses major challenges to parents and their children seeking advice for the best options (52), illustrated mathematically by a multi-state model analysis of a large trial cohort (52). The developmental framework of childhood and adolescence makes decisions at different developmental windows and ages, influenced by stages of brain growth and pubertal maturation, physical characteristics of skeletal growth, as well as social and neuro-psychological maturational competence. This is made more complex by the child's and their family's experience of visual-impairment and/or the clinical complexities of panhypopituitarism. The multi-disciplinary considerations are heavily determined by which specialists are involved in the discussion with the family, their experiences and their own beliefs (53) A model is proposed in Figure 5.

To date, there is no formal scoring system where the potential for, and importance of, preservation of visual, endocrine or neuro-behavioural outcomes can be weighed against each other with different treatment approaches. This complexity is a major challenge to communication between physician and the child and family seeking advice on the “best interests” for the child in this disease. It is difficult for parents to find an equitable emotional balance between their perception of risks of mortality vs. risk of lifelong disability for the wide variety of outcomes for their child. Mortality is a categorical risk at a moment of diagnosis, feared by the parent, whilst disability is a qualitative risk over a lifetime. It is frequently a shared experience by the developing individual and their “supporters and advocates”.

## What are the trial questions under current study?

Developments in the application of novel technology to this disease are occurring. There has been an explosion in biological understanding of tumor tissue biology in childhood. There is a global emphasis now placed upon the need to optimise diagnostic pathways for children with cancers as part of the WHO Cure All Strategy (54). This strategy seeks to influence health systems from all economic categories of countries to level up outcomes for children with cancer globally, justified by a health economic capacity to triple the impact of any investment on health outcomes (55). For this to be realised in brain tumors, a strong focus on reducing neuro-disability with its economic consequences is required. OPHGs represent one of the commonest groups of tumors with clearly defined disabilities of acquired vision loss, endocrine, neurological and developmental deficits with lifelong consequences. Consequently, they offer opportunities for risk stratified approaches to new therapies seeking to reduce disability outcomes.

## What are the outstanding questions from patients and families and health care providers?

Experience with the HeadSmart programme in the UK identified the impact of raising awareness amongst the public and professional communities to accelerate diagnosis of childhood brain tumor within a national health system (3, 56). Similar projects have now been launched in several countries. In high income countries (HICs), OPHG have been identified as one of the most common treatable cause of vision loss in children (57), justifying special consideration for accelerating diagnosis. The neuro-paediatricians diagnose and manage OPHGs with geneticists, neuro-ophthalmologists, brain imaging specialists and endocrinologists, who all work with specialist teams to screen, diagnose and manage the neurotoxic and endocrine consequences of these tumors. As time passes and the child becomes an adult, the need for lifelong rehabilitative neurobehavioural and endocrine/follow-on clinics to transition successfully into adult services. System models exist but their further development (58) requires the health economic data to justify their incorporation into adult service models of public or private health service commissioners or insurers (59).

## How will the emerging biological evidence influence current and future trials?

The past 2 decades has seen the biology of pilocytic astrocytoma (PA) explored in detail. Nearly 100% of pilocytic astrocytoma have mutations involving the MAPK/ERK signalling pathway regulation, where BRAF kinase alterations are considered to be the characteristic hallmark. The most common rearrangement is a fusion between KIAA 1549 and BRAF genes which occurs in 70% of PAs; the next most common are inactivating NF1 alterations and oncogenic BRAF^V600E^. Others reported less frequently are other BRAF fusions, FGFR1 mutations or fusions, NTRK2 fusions and oncogenic KRAS mutations. They all activate the MAPK/ERK pathway, making PA a single pathway disease, ideal for therapeutic targeting (5).

### Targeting the NF1 gene

A recent review identified that clinical examination of patients combined with molecular analyses is beginning to reveal *NF1* genotype-phenotype correlations - such findings will help define novel functions of neurofibromin, its interactions with the tissue microenvironment and hormonal milieu. Sustained research, driven by access to patient samples for the development of patient and cell-specific models reflecting the human disease will drive cellular pathway analysis and the identification of therapeutic targets and biomarkers suitable for pre-clinical testing. A range of novel strategies are already under consideration including synthetic lethal screening (using CRISPR libraries), immune profiling for immunotherapy and generation of novel biomarkers for NF1-associated tumors. Gene therapy approaches focus on antisense oligonucleotides (ASOs) and nonsense suppression, whereas potential correction of mutations *via* gene editing offers a possibility of restoring endogenous *NF1* gene function, thereby providing a long-term solution for NF1 patients (60).

### New trials of therapies

Drugs targeting MEK inhibition (MEKi) have been selected for testing in NF1- and BRAF-altered paediatric low-grade gliomas (pLGG) and for PAs in particular. The MEKi Selumetinib showed promising results in phase I and II trials (61–64). Similarly, the MEKi Trametinib is under trial for recurrent NF1-associated and BRAF-fusion pLGGs (65). Another trial (NCT 03871257) is investigating Selumetinib in conjunction with vincristine/carboplatin in a front line setting for NF1-mutant pLGG. The European LOGGIC trial will be the first prospective randomised 2-arm study of pLGGs harbouring an active RAF mutation, comparing an oral pan-RAF inhibitor tovorafinib (DAY101) vs. standard of care carboplatin/vincristine or vinblastine monotherapy as first line treatment (65, 66). Most recently, results of the prospective randomized phase II trial (NCT02684058) of the combination of a BRAF inhibitor dabrafenib (dab) and the MEKi trametinib (tram) as first line therapy for BRAF^v600E^-mutant pLGG identified that the “dab + tram” combination increased overall response rate and clinical benefit rate and prolongs progression free survival when compared with carboplatin and vincristine. These encouraging results and the tolerable safety profile suggest that “dab + tram” may be a promising first-line systemic treatment option for this patient population.

### A preliminary consensus for treatment selection

A recent proposal for a consensus mapped the molecular relationship between the tumor's anatomical location, the age of the child and histological characteristics of the tumor tissue. They identified 3 groups and justified clinical approaches ranging from adopting either a conservative approach, or being pro-active or identifying cases justifying more aggressive approaches. They did not map their stratifying factors onto late neurological, endocrine or neuropsychological/ behavioural outcomes or indeed data concerning pre-diagnostic intervals. The consensus therefore is tumor-centered and not patient-centered and may be considered simplistic as it disregards the clinical experience of survivorship, summarised in this review (67). Despite this criticism, the biological research that has identified the wide range of molecular targets offers real hope of effective therapies that are in the process of translation through clinical trials. It is imperative, at this time, that the missing elements of this consensus are given careful consideration as not all problems will be solved by the new drugs being developed for many reasons. Furthermore, health services and translational research directed at neuroprotection already may offer opportunities to apply novel approaches for minimising adverse consequences affecting survivorship. Evidence already exists which demonstrates the potential for the role of bevacizumab in preserving and improving visual outcomes at the time of tumor progression (68); topically applied nerve growth factor has been shown to restore optic nerve function (69) in children with OPHG; preliminary research is reported where brain stem-cell therapy is being investigated for brain injury repair (70) as well as evidence of rising health service awareness of the need for early symptom awareness and specific services to support children after acquired brain injury (58, 71).

### What are the anticipated developments?

These trials of new therapies are specifying clinical outcomes such as vision assessments as a primary outcome measure(s). Based upon the new reports of endocrine and neuro-behavioural outcomes for children with OPHG and the uncertainties of how to select patients for treatment vs. observation, we conclude that further studies are needed to dissect the impact of tumor progression vs. consequences of treatment on these additional health outcomes (16). A trial design selecting patients for randomisation between initial observation vs. initial therapy is justified by the existing uncertainty for when to treat or observe children using vision loss and endocrine outcomes as primary outcomes. It could be highly informative and integrated with trials of new tumor agents as part of pre-treatment registration. Such an approach would generate valuable evidence to reduce the current levels of uncertainty as to who to treat and who to observe. Taken together, the potential for these translational trials and health system interventions raise hope that it will be possible to reduce the impact of OPHG upon the late consequences of this disease and its treatment (72).

## Anticipated developments

• *Accelerating diagnosis* by raising awareness of the risk and the classical neuroendocrine and intracranial pressure presentations of OPHG as well research to target populations for screening or surveillance. The opportunity exists to use a combination of NF status and clinical growth, visual, developmental biomarkers for case selection for vision testing or scanning. If applied successfully it could tackle the prolonged pre-diagnostic interval that is a characteristic feature of tumors arising around the middle of the brain and optic tracts (3, 73). This approach is justified by clinical and legal arguments used to justify compensatory awards to individuals identified as suffering additional observed disability as a consequence of diagnostic delay (74).
• S*tandardised approaches to visual acuity testing* will permit more reliable assessments of visual performance as part of treatment selection and outcomes assessments in practice and trials (46, 47, 75).
• *Innovation in brain and retinal imaging* of OPHG has produced a *refined anatomical classification* of OPHG with more detailed functional descriptors (42). Diffusion Tensor Imaging is being used to explore the possibility of *predictive scoring system for vision loss* (*77*)*. Optical coherence tomography* offers measurements of *retinal fibre layer thickness* as an objective measure of nerve loss as part of visual outcome monitoring, as well as the opportunity to understand the relationship between retinal nerve injury and tumor location, tumor size and growth across the optic tracts (43).
• *Introduction of risk stratification for early and developing neuroendocrine and neurodevelopmental* deficits which combine to emerge as so-called late consequences as part of cost-benefits of treatment vs. observation decision making within clinical trials and outcome studies (26).
• The *role of surgery is under scrutiny* for tumors arising in different locations. Optic nerve tumors are no longer considered to be a risk for chiasmatic extension. Surgeons are working toward*s a consensus for attempted surgical resection* as part of safe surgery approaches. Such strategies need to offer low risks of endocrine and neurological/ neurodevelopmental toxicity (14) and should be followed by targeted rehabilitation.
• *Proton therapy* offers reduced risks by enhanced precision of radiation field planning. Research into the lifelong benefits and risks is needed (37)
• *Trials of novel tumor targeted agents* used alone or in combination offer more precisely biologically targeted treatments aimed at changing the damaging effects of tumors on neuronal and hypothalamic functioning (77). Research into the relationship between tumor shrinkage, vision preservation and neuro-endocrine outcomes is needed.
• *Research targeting the biology of the tumor micro-environment in sporadic and NF1 associated tumors,* given the developmental features governing tumor growth and senescence (5, 78, 79). Research into the interaction between tumor cells and neuronal functioning (80) or immune mechanisms that may influence tumor microenvironment in all stages of tumor development (81–83) is required to explain clinical phenomena.
• *Treating brain injury with neuronal protection or restorative therapies* (69, 70).
